# Traumatic Takotsubo Cardiomyopathy in a Patient with Extensive Coronary Artery Disease

**DOI:** 10.1155/2019/7270426

**Published:** 2019-06-12

**Authors:** Abagayle E. Renko, Warren C. Doyle, Paul W. Sokoloski

**Affiliations:** Penn State Milton S. Hershey Medical Center, Department of Emergency Medicine, Hershey, PA 17033, USA

## Abstract

Takotsubo Cardiomyopathy (TCM) should be considered in the differential diagnosis for patients with cardiovascular symptoms not only following emotional trauma but also following motor vehicle accidents. A 45-year-old woman presented with chest pain following a motor vehicle accident. While she had an elevated troponin level and an extensive history of cardiac disease, her electrocardiogram was normal. Echocardiogram, however, demonstrated transiently reduced left ventricular systolic function with mid to apical hypokinesis consistent with TCM. We emphasize the use of a diagnostic score and point of care focused cardiac ultrasound (FOCUS) to expedite the recognition, evaluation, and treatment of suspected TCM in an Emergency Department setting.

## 1. Introduction

Takotsubo Cardiomyopathy (TCM), also known as stress-induced cardiomyopathy or left ventricular apical ballooning syndrome, was first described by Japanese physicians in 1990 [1]. Named after a Japanese pot used to trap octopuses, this syndrome typically presents in postmenopausal women with symptomatology, EKG changes, and cardiac enzymes indistinguishable from that of an acute myocardial infarction [2, 3]. In the absence of any abnormalities identified on coronary angiography or coronary computed tomography angiography (CCTA), echography typically confirms the diagnosis demonstrating transient hypokinesis, akinesis, or dyskinesis of left ventricular midsegments with or without apical involvement [3–5]. Exposure to an emotional or physical stressor typically incites TCM, with reception of bad news persisting as its most common and recognizable trigger. However, numerous other inciting events have been reported including amphetamine ingestion, alcohol intoxication, diabetic ketoacidosis, status asthmaticus, severe vomiting, thyrotoxicosis, urosepsis, influenza A infection, seizure, cesarean section, chemotherapy, radioactive iodine, hyponatremia, a snake bite, a lightning strike, near drowning, and exercise [6–23].

Though initially thought to be a rare phenomenon, according to recent data, TCM is estimated to account for 1% to 2% of all cases of suspected myocardial infarction, with a peak incidence during summertime [24, 25]. Many patients have a history of smoking, alcohol abuse, anxiety states, or hyperlipidemia [25]. While the pathophysiology of TCM remains largely unknown, it has been hypothesized that sudden and severe emotional or physical stress activates central autonomic neurons with estrogen receptors which markedly increases sympathetic neuronal and adrenomedullary hormonal outflow. Epinephrine binds to adrenoreceptors, thus causing contraction of blood vessels and subsequent increased systemic blood pressure and afterload, while inducing catecholamine toxicity on cardiomyocytes within the heart itself. This toxicity is manifested in a heterogeneous manner, with myocardial cell rupture occurring in areas with higher apical expression of adrenoreceptors. It is thought that estrogen loss exaggerates these central neuron and cardiomyocyte responses, thus predisposing postmenopausal women to TCM [26].

We report a case of a young female patient who presented to the Emergency Department with chest, neck, and back pain following a motor vehicle accident, who was later found to have Takotsubo Cardiomyopathy.

## 2. Case Presentation

A 45-year-old obese postmenopausal female with a history of coronary artery disease (CAD), hypertension, hyperlipidemia, diabetes, and chronic angina was brought to the Emergency Department midsummer after a motor vehicle accident (MVA) in which she was the restrained driver. While stationary, she was struck by another vehicle from behind at a high rate of speed with immediate airbag deployment. Though the patient did not lose consciousness at the scene and had self-extricated prior to EMS arrival, she requested hospital transport for further evaluation of chest, neck, and back pain. On initial evaluation, the patient complained of chest pain primarily across her left chest and shoulder in a seatbelt-type distribution, though she did also endorse some bilateral chest wall pain, diffuse midline neck pain, and low back pain.

In addition to her cardiac risk factors, her medical history was significant for anxiety, gastroesophageal reflux disease (GERD), migraines, and fibromyalgia. Her home medications included escitalopram, buspirone, pantoprazole, ranitidine, aspirin, clopidogrel, evolocumab, and isosorbide mononitrate. She had an extensive surgical history including a total abdominal hysterectomy, bilateral salpingo-oophorectomy, coronary artery bypass grafting (CABG), and placement of coronary stents. She denied current use of tobacco, alcohol, or illicit drugs. She had just been admitted to the hospital for unstable angina a few months prior, at which time cardiac catheterization was performed, demonstrating three patent bypass grafts: right internal mammary artery (RIMA) to left anterior descending artery (LAD), left internal mammary artery (LIMA) to obtuse marginal artery, and a saphenous vein graft from the proximal aorta to the proximal right coronary artery (RCA). One saphenous vein graft from the posterior descending artery (PDA) to the posterolateral ventricular artery segment was occluded, though this was not a new finding as it was first discovered to be occluded several years ago. Both previously placed drug-eluding stents in the second marginal and left circumflex arteries were widely patent. The catheterization also demonstrated severely diseased and diffusely calcified coronary arteries, with 95% occlusion in the proximal LAD and 100% occlusion in both the first marginal artery and the mid to distal RCA. Of note, her stenosis had worsened since her last catheterization one year earlier, which showed 70% occlusion in the mid-LAD, 90% occlusion in the distal LAD, and 90% occlusion in the mid to distal RCA. No intervention was performed during either catheterization procedure.

Physical exam revealed that the patient was in notable distress and had diffuse midline spinal and paraspinal tenderness, as well as chest wall tenderness. Her pulse was 100/minute, temperature 37.6°C, blood pressure 157/119 mmHg, respiratory rate 16 breaths/min, and SpO_2_ 99% on room air. Chest radiographs and head computed tomography (CT) scans were unremarkable, and the aorta had a normal course and caliber on thorax CT scan. Electrocardiogram (EKG) was also unremarkable, without any ST depression or elevation, T wave inversions, or QTc prolongation noted. Laboratory workup revealed an elevated troponin-T level at 0.564 [normal <0.010]. The patient was not hyponatremic. Three hours later, her troponin-T level decreased to 0.459. BNP, CK, and lactate levels were not obtained.

**Figure 1:**
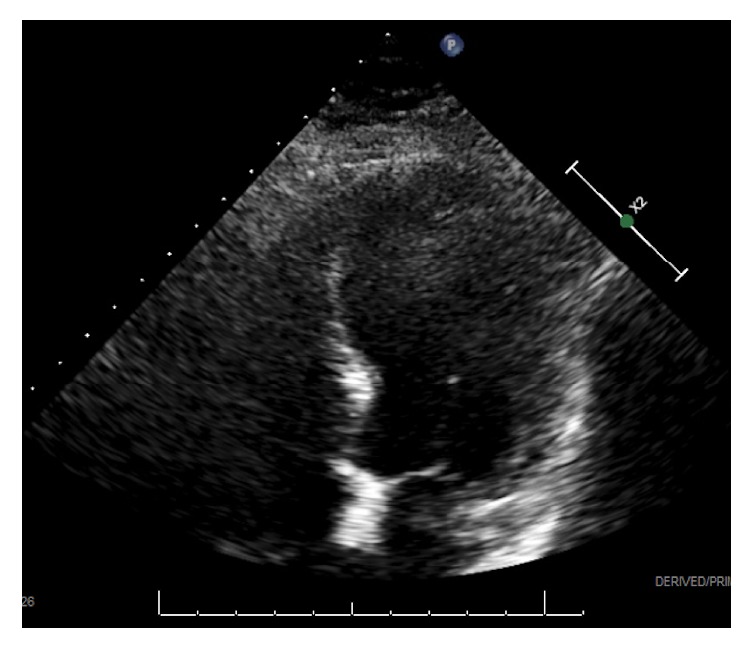
Apical four-chamber view demonstrating mid to apical ballooning of left ventricle.

Cardiac contusion secondary to blunt chest trauma from the MVA was suspected as the initial diagnosis. The patient was admitted to the acute cardiology service and underwent a transthoracic echocardiogram the following morning, which demonstrated reduced systolic function with an ejection fraction of 35%, diffuse areas of mid to apical hypokinesis, and hyperkinesis in the anterior and inferior basal segments (Figure 1). Previous echocardiograms completed three months and one year prior to this episode did not show these abnormalities; those studies lacked wall motion abnormalities and demonstrated normal systolic function with ejection fractions of 60% and 65%, respectively. Thus, these new wall motion abnormalities and reduced systolic function in conjunction with elevated cardiac enzymes, a normal electrocardiogram, and the patient's clinical presentation were consistent with a diagnosis of stress-induced cardiomyopathy.

She was started on a beta blocker and discharged the next day. A follow-up echocardiogram four months later demonstrated return of systolic function with an improved ejection fraction of 55% and normal wall motion, indicating complete resolution.

## 3. Discussion

Takotsubo Cardiomyopathy (TCM), also known as stress-induced cardiomyopathy, is a condition that typically presents in postmenopausal women with chest pain and EKG changes often indistinguishable from that of an acute myocardial infarction. To our knowledge, this is only the second reported instance after a motor vehicle accident and the first reported instance in an individual with such extensive preexisting coronary artery disease.

Though Mayo Clinic's initially published and still widely accepted diagnostic criteria for TCM include the absence of obstructive angiographic coronary disease or angiographic evidence of acute plaque rupture, the institution has since modified its criteria to refute the presence of CAD as exclusion criteria [4, 28]. Several other groups have also since published their own modified criteria [29–34]. In an attempt to achieve a worldwide diagnostic consensus, international experts recently collaborated and published the International Takotsubo Diagnostic Criteria (Table 1) to improve identification and stratification of TCM [27]. Following the diagnostic algorithm suggested by the International Expert Consensus Document, providers are encouraged to calculate an InterTAK Diagnostic Score (Table 2) for any patient without ST elevation on EKG; if the patient scores greater than 70 points, the authors suggest proceeding directly to a transthoracic echocardiogram (TTE). However, we argue that if TTE is not readily available, a point of care focused cardiac ultrasound (FOCUS) should be sufficient to diagnose TCM in the Emergency Department if features such as systolic mid to apical hypokinesis of the left ventricle and appropriate systolic contraction of basal segments near the atrioventricular septum are identified [5].

It has been hypothesized that mid-LAD plaque rupture followed by rapid lysis could cause transient ischemia and myocardial stunning that may mimic TCM, though ultrasound and more invasive strategies have failed to identify any evidence of ruptured plaque in the majority of patients with suspected TCM [35–37]. Cases have also been reported where TCM coexisted with an acute coronary syndrome (ACS), though the patients described in those cases had identifiable EKG changes [38]. In a patient with suspected TCM with coexisting and significant CAD, one would ideally be able to carefully compare coronary angiography with biplane ventriculography in similar views to identify any perfusion or contraction mismatches [38, 39]. Invasive intracoronary Doppler would also suggest a diagnosis of TCM if short diastolic deceleration times and decreased coronary flow velocity reserves (CFR) were observed [40]. However, less invasive strategies may be initially preferred in patients like ours who present with elevated cardiac biomarkers yet lack acute EKG changes and have documented access difficulty due to prior repeated catheterizations.

To further differentiate TCM from an ACS utilizing a noninvasive ultrasound, one should not only look for wall motion abnormalities but also attempt to identify any right ventricular extension or systolic anterior motion of the mitral valve; these abnormalities would favor the diagnosis of TCM, rather than an anterior wall myocardial infarction (MI) [41, 42]. Furthermore, contrast echocardiography should demonstrate normal myocardial perfusion in the akinetic apical area in a patient with TCM, while one would expect impaired flow with an anterior wall MI [43]. Less invasive Doppler flow strategies can also be utilized to assess the patency and flow of the LAD and differentiate between these conditions, for an undetectable distal LAD velocity in the presence of apical left ventricular wall motion abnormalities is suggestive of ACS due to LAD occlusion [44]. By contrast, detection of decreased CFR would support a diagnosis of TCM, for CFR is transiently impaired in the acute phase of TCM [45]. This data would not be available during an initial patient assessment in the Emergency Department, however, as it must be obtained during a TTE study.

Though our patient's clinical course proved to be fairly benign, her diagnosis was delayed until wall motion abnormalities were detected on an echocardiogram several hours following her initial presentation. If this patient's InterTAK Diagnostic Score had been calculated after an EKG was performed and found to be normal, TCM could have been suspected and a diagnosis could have likely been made earlier in her course through a bedside FOCUS in the Emergency Department. Any subsequent echocardiograms could have been performed not to diagnose but rather to monitor for potential complications of TCM such as mitral regurgitation, left ventricular free wall rupture, and thrombus formation [34, 46–48]. BNP levels also should have been obtained, for serial monitoring of this biomarker allows for continued noninvasive assessment of one's improvement in left ventricular dysfunction over time [49, 50].

Treatment of TCM is debated in reported literature, though beta blockers have remained the mainstay of treatment largely due to the accepted role of catecholamines in its pathophysiology. Interestingly, however, the Takotsubo Registry reports that angiotensin-converting enzyme (ACE) inhibitors or angiotensin receptor blockers (ARBs) have been associated with improved survival of TCM patients in comparison to those treated with beta blockers alone [51]. Maintaining a high index of suspicion for TCM is important to ensure not only that adequate treatment is given, but also that potentially harmful interventions such as fibrinolytic therapy are avoided.

## 4. Conclusion

Takotsubo Cardiomyopathy (TCM) accounts for a notable portion of cardiac enzyme-elevated acute coronary syndromes and thus deserves more widespread recognition amongst Emergency Medicine physicians. It should be included in one's differential diagnosis in any patient presenting after a stressful event with chest pain and associated cardiac enzyme elevations. While timely echocardiography and/or cardiac catheterization have been the mainstay of evaluation for this condition, calculation of a diagnostic score and evaluation with point of care ultrasonography can sufficiently make an ambulatory diagnosis in patients with extensive preexisting coronary artery disease and no acute EKG changes. Following consistent improvements in biomarkers (troponins, BNP) and absence of complications on echocardiography, patients can often be safely discharged on combination antihypertensive therapy with scheduled follow-up in an outpatient setting.

## Figures and Tables

**Table 1:** International Takotsubo Diagnostic Criteria (InterTAK Diagnostic Criteria) [27].

1.	Patients show transient^a^ left ventricular dysfunction (hypokinesia, akinesia, or dyskinesia) presenting as apical ballooning or midventricular, basal, or focal wall motion abnormalities. Right ventricular involvement can be present. Besides these regional wall motion patterns, transitions between all types can exist. The regional wall motion abnormality usually extends beyond a single epicardial vascular distribution; however, rare cases can exist where the regional wall motion abnormality is present in the subtended myocardial territory of a single coronary artery (focal TTS).^b^

2.	An emotional, physical, or combined trigger can precede the takotsubo syndrome event, but this is not obligatory.

3.	Neurologic disorders (e.g. subarachnoid haemorrhage, stroke/transient ischaemic attack, or seizures) as well as pheochromocytoma may serve as triggers for takotsubo syndrome.

4.	New ECG abnormalities are present (ST-segment elevation, ST-segment depression, T-wave inversion, and QTc prolongation); however, rare cases exist without any ECG changes.

5.	Levels of cardiac biomarkers (troponin and creatine kinase) are moderately elevated in most cases; significant elevation of brain natriuretic peptide is common.

6.	Significant coronary artery disease is not a contradiction in takotsubo syndrome.

7.	Patients have no evidence of infectious myocarditis.^b^

8.	Postmenopausal women are predominantly affected.

^a^Wall motion abnormalities may remain for a prolonged period of time or documentation of recovery may not be possible. For example, death before evidence of recovery is captured.

^b^Cardiac magnetic resonance imaging is recommended to exclude infectious myocarditis and diagnosis confirmation of takotsubo syndrome.

**Table 2:** InterTAK Diagnostic Score Point System [27].

Risk Factor	Points Assigned
Female sex	25 points
Emotional stress	24 points
Physical stress	13 points
No ST segment depression, except in lead aVR	12 points
Psychiatric disorders	11 points
Neurologic disorders	9 points
QTc prolongation	6 points

## References

[1] Sato H., Tateishi H., Uchida T., Kodama K., Haze K., Hon M. (1990). Takotsubo-type cardiomyopathy due to multivessel spasm. *Clinical Aspect of Myocardial Injury: From Ischemia to Heart Failure*.

[2] Fazal I. A., Alfakih K., Walsh J. T. (2007). Takotsubo cardiomyopathy. *Journal of the Royal Society of Medicine*.

[3] Yoon D. Y., Dole A., Gonsalves L. (2011). Takotsubo (stress-induced) cardiomyopathy in post-menopausal women. *Psychosomatics*.

[4] Prasad A., Lerman A., Rihal C. S. (2008). Apical ballooning syndrome (Tako-Tsubo or stress cardiomyopathy): a mimic of acute myocardial infarction. *American Heart Journal *.

[5] Bossone E., Lyon A., Citro R. (2014). Takotsubo cardiomyopathy: an integrated multi-imaging approach. *European Heart Journal - Cardiovascular Imaging*.

[6] Meigh K., Caja M., Sharon M. (2018). Takotsubo cardiomyopathy in the emergency department: a FOCUS heart breaker. *The Western Journal of Emergency Medicine*.

[7] Toce M. S., Farias M., Bruccoleri R., Brown D. W., Burns M. M. (2017). A case report of reversible takotsubo cardiomyopathy after amphetamine/dextroamphetamine ingestion in a 15-year-old adolescent girl. *Journal of Pediatrics*.

[8] Mitchell S. A., Crone R. A. (2006). Takotsubo cardiomyopathy: a case report. *Journal of the American Society of Echocardiography*.

[9] Meyers J. H., Hirsch I. B. (2017). Takotsubo cardiomyopathy in association with DKA in a blind pump patient. *AACE Clinical Case Reports*.

[10] Kotsiou O. S., Douras A., Makris D., Mpaka N., Gourgoulianis K. I. (2017). Takotsubo cardiomyopathy: a known unknown foe of asthma. *Journal of Asthma & Allergy Educators*.

[11] Mazen A., Hernandez R. A., Bach D. S. (2008). Takotsubo cardiomyopathy triggered by severe vomiting. *American Journal of Medicine*.

[12] Eliades M., El-Maouche D., Choudhary C., Zinsmeister B., Burman K. D. (2014). Takotsubo cardiomyopathy associated with thyrotoxicosis: a case report and review of the literature. *Thyroid*.

[13] Omar H. R., Mangar D., Camporesi E. M. (2014). Urosepsis-induced takotsubo. *The American Journal of Emergency Medicine*.

[14] Taniguchi K., Takashima S., Iida R. (2017). Takotsubo cardiomyopathy caused by acute respiratory stress from extubation: a case report. *Medicine*.

[15] Stöllberger C., Wegner C., Finsterer J. (2011). Seizure-associated Takotsubo cardiomyopathy. *Epilepsia*.

[16] Citro R., Lyon A., Arbustini E. (2018). Takotsubo syndrome after cesarean section: rare but possible. *Journal of the American College of Cardiology*.

[17] Malley T., Watson E. (2016). A case of Takotsubo cardiomyopathy after chemotherapy. *Oxford Medical Case Reports*.

[18] Dimakopoulou A., Vithian K., Gannon D., Harkness A. (2015). Stress cardiomyopathy (Takotsubo) following radioactive iodine therapy. *Endocrinology, Diabetes & Metabolism Case Reports*.

[19] Murase K., Takagi K. (2012). Takotsubo cardiomyopathy in a snake bite victim: a case report. *Pan African Medical Journal*.

[20] Dundon B. K., Puri R., Leong D. P., Worthley M. I. (2008). Takotsubo cardiomyopathy following lightning strike. *Emergency Medicine Journal*.

[21] Citro R., Patella M. M., Bossone E., Maione A., Provenza G., Gregorio G. (2008). Near-drowning syndrome: a possible trigger of tako-tsubo cardiomyopathy. *Journal of Cardiovascular Medicine*.

[22] Chams S., El Sayegh S., Hamdon M., Kumar S., Kulairi Z. (2018). Zumba-induced Takotsubo cardiomyopathy: a case report. *Journal of Medical Case Reports*.

[23] De Giorgi A., Fabbian F., Tiseo R. (2014). Takotsubo cardiomyopathy and endocrine disorders: a mini-review of case reports. *The American Journal of Emergency Medicine*.

[24] Kurowski V., Kaiser A., Von Hof K. (2007). Apical and midventricular transient left ventricular dysfunction syndrome (Tako-Tsubo cardiomyopathy): frequency, mechanisms, and prognosis. *CHEST*.

[25] Deshmukh A., Kumar G., Pant S., Rihal C., Murugiah K., Mehta J. L. (2012). Prevalence of Takotsubo cardiomyopathy in the United States. *American Heart Journal*.

[26] Akashi Y. J., Goldstein D. S., Barbara G., Ueyama T. (2008). Takotsubo cardiomyopathy a new form of acute, reversible heart failure. *Circulation*.

[27] Ghadri J., Wittstein I. S., Prasad A. (2018). International expert consensus document on takotsubo syndrome (Part I): clinical characteristics, diagnostic criteria, and pathophysiology. *European Heart Journal*.

[28] Scantlebury D. C., Prasad A., Prasad A. (2014). Diagnosis of takotsubo cardiomyopathy – Mayo Clinic criteria. *Circulation Journal*.

[29] Kawai S., Kitabatake A., Tomoike H. (2007). Guidelines for diagnosis of takotsubo (Ampulla) cardiomyopathy. *Circulation Journal*.

[30] Schultz T., Shao Y., Redfors B. (2012). Stress-induced cardiomyopathy in Sweden: evidence for different ethnic predisposition and altered cardio-circulatory status. *Cardiology*.

[31] Wittstein I. S. (2012). Stress cardiomyopathy: a syndrome of catecholamine-mediated myocardial stunning?. *Cellular and Molecular Neurobiology*.

[32] Parodi G., Citro R., Bellandi B., Provenza G., Marrani M., Bossone E. (2014). Revised clinical diagnostic criteria for Tako-tsubo syndrome: the Tako-tsubo Italian Network proposal. *International Journal of Cardiology*.

[33] Madias J. E. (2014). Why the current diagnostic criteria of Takotsubo syndrome are outmoded: a proposal for new criteria. *International Journal of Cardiology*.

[34] Lyon A. R., Bossone E., Schneider B. (2015). Current state of knowledge on takotsubo syndrome: a position statement from the taskforce on takotsubo syndrome of the heart failure Association of the European Society of Cardiology. *European Journal of Heart Failure*.

[35] Delgado G. A., Truesdell A. G., Kirchner R. M. (2011). An angiographic and intravascular ultrasound study of the left anterior descending coronary artery in takotsubo cardiomyopathy. *American Journal of Cardiology*.

[36] Pawłowski T., Mintz G. S., Kulawik T., Gil R. J. (2010). Virtual histology intravascular ultrasound evaluation of the left anterior descending coronary artery in patients with transient left ventricular ballooning syndrome. *Kardiologia Polska*.

[37] Eitel I., Stiermaier T., Graf T. (2016). Optical coherence tomography to evaluate plaque burden and morphology in patients with takotsubo syndrome. *Journal of the American Heart Association*.

[38] Haghi D., Papavassiliu T., Hamm K., Kaden J. J., Borggrefe M., Suselbeck T. (2007). Coronary artery disease in Takotsubo cardiomyopathy. *Circulation Journal*.

[39] Patel S. M., Lennon R. J., Prasad A. (2012). Regional wall motion abnormality in apical ballooning syndrome (Takotsubo/stress cardiomyopathy): importance of biplane left ventriculography for differentiating from spontaneously aborted anterior myocardial infarction. *The International Journal of Cardiovascular Imaging*.

[40] Kume T., Akasaka T., Kawamoto T. (2005). Assessment of coronary microcirculation in patients with takotsubo-like left ventricular dysfunction. *Circulation Journal*.

[41] Citro R., Piscione F., Parodi G., Salerno-Uriarte J., Bossone E. (2013). Role of echocardiography in takotsubo cardiomyopathy. *Heart Failure Clinics*.

[42] Mejía-Rentería H. D., Núñez-Gil I. J. (2016). Takotsubo syndrome: advances in the understanding and management of an enigmatic stress cardiomyopathy. *World Journal of Cardiology*.

[43] Lee J., Kim J. (2011). Stress-induced cardiomyopathy: the role of echocardiography. *Journal of Cardiovascular Ultrasound*.

[44] Sharif D., Sharif-Rasslan A., Shahla C., Abinader E. G. (2010). Detection of severe left anterior descending coronary artery stenosis by transthoracic evaluation of resting coronary flow velocity dynamics. *Heart International*.

[45] Meimoun P., Malaquin D., Benali T., Boulanger J., Zemir H., Tribouilloy C. (2009). Transient impairment of coronary flow reserve in tako-tsubo cardiomyopathy is related to left ventricular systolic parameters. *European Heart Journal - Cardiovascular Imaging*.

[46] Ishida T., Yasu T., Arao K., Kawakami M., Saito M. (2005). Images in cardiovascular medicine. Bedside diagnosis of cardiac rupture by contrast echocardiography. *Circulation*.

[47] Haghi D., Papavassiliu T., Heggemann F., Kaden J. J., Borggrefe M., Suselbeck T. (2008). Incidence and clinical significance of left ventricular thrombus in tako-tsubo cardiomyopathy assessed with echocardiography. *QJM: An International Journal of Medicine*.

[48] Buchholz S., Ward M. R., Bhindi R., Nelson G. I. C., Figtree G. A., Grieve S. M. (2010). Cardiac thrombi in stress (tako-tsubo) cardiomyopathy: more than an apical issue?. *Mayo Clinic Proceedings*.

[49] Nguyen T. H., Neil C. J., Sverdlov A. L. (2011). N-terminal pro-brain natriuretic protein levels in takotsubo cardiomyopathy. *American Journal of Cardiology*.

[50] Akashi Y. J., Musha H., Nakazawa K., Miyake F. (2004). Plasma brain natriuretic peptide in takotsubo cardiomyopathy. *QJM: Monthly Journal of the Association of Physicians*.

[51] Templin C., Ghadri J. R., Diekmann J. (2015). Clinical features and outcomes of takotsubo (stress) cardiomyopathy. *The New England Journal of Medicine*.

